# Effects of GLP-1 receptor agonists on asprosin levels in normal weight or overweight/obesity patients with type 2 diabetes mellitus

**DOI:** 10.1097/MD.0000000000031334

**Published:** 2022-10-28

**Authors:** Chenggang Dai, Weifeng Zhu

**Affiliations:** a Guangzhou University of Chinese Medicine, Guangzhong, China; b Endocrinology Department, Guangzhou Development District Hospital, Guangzhou, China.

**Keywords:** asprosin, liraglutide, type 2 diabetes

## Abstract

Asprosin is a newly identified adipokine with glucose-raising and appetite-enhancing effects which acts differently from the known hepatic glucose utilization pathway. This study investigated changes in serum asprosin levels in normal weight or overweight/obese liraglutide-treated patients with type 2 diabetes (T2DM). This study is a non-randomized, prospective observational study. The metabolic parameters and asprosin levels were compared between 90 people with T2DM and 66 people who had normal glucose tolerance (NGT). During the treatment phase, only T2DM patients were given liraglutide at doses of 0.6 mg/d for the first 2 weeks, 1.2 mg/d for the subsequent 4 weeks, and 1.8 mg/d for the following 16 weeks. T2DM patients were separated into a normal weight group and an overweight/obesity group to compare changes in asprosin and parameters pre- and post-treatment. The T2DM group had significantly higher fasting asprosin and 2h-postprandial asprosin levels than the NGT group (all *P* < .001). Fasting asprosin and postprandial asprosin positively correlated with BMI, 2hPG, HbA1c, TG, and HOMA-IR, and negatively correlated with HDL-C in both the T2DM and NGT groups. Asprosin levels decreased after liraglutide treatment in both normal and overweight/obesity T2DM groups (all *P* < .001), with significantly reduced body weight and BMI in overweight/obese T2DM patients (all *P* < .001). Fasting and postprandial serum asprosin concentrations are higher in T2DM patients compared to normal glucose controls. Fasting and postprandial asprosin positively correlated with BMI, 2hPG, HbA1c, TG, and HOMA-IR and negatively correlated with HDL-C in all participants. Liraglutide lowers asprosin levels in T2DM patients and can reduce weight and BMI in overweight or obese type 2 diabetics.

## 1. Introduction

The prevalence of type 2 diabetes (T2DM) is rising globally resulting in serious complications that cause severe psychological and physical distress and place a considerable burden on healthcare systems. Most people with T2DM are overweight or obese,^[1]^ which can lead to insulin resistance (IR), beta-cell dysfunction, and chronic systemic inflammation, making blood glucose control more difficult.^[2–4]^ Therefore, when choosing T2DM medication, both blood glucose and body weight need to be taken into account.

Asprosin is an adipokine identified 6 years ago. It is a cleaved fragment of the C-terminus of profibrillin, which is secreted by white adipocytes and has gluconeogenic and appetite-enhancing effects that increase with fasting and decrease with feeding.^[5]^ Asprosin levels are significantly elevated in insulin-resistant states. Asprosin has a glycemic pathway that differs from the currently known classical hepatic glucose utilization pathway,^[6]^ thus providing new ideas and approaches for T2DM treatment by intervening in the asprosin glucagon pathway.

Glucagon-like peptide 1 receptor agonists (GLP-1RAs) are ideal for patients with overweight T2DM because they control blood glucose and promote weight loss.^[7,8]^ Liraglutide is a representative medication effectively used to treat overweight T2DM patients and has a low risk of hypoglycemia.^[9]^

Our experiment compared serum asprosin levels between NGT and T2DM people and investigated the correlation between asprosin and some important parameters. Then, we observed the effect of liraglutide on asprosin in normal weight or overweight/obesity patients with T2DM to identify new targets for T2DM treatment.

This study investigated the effect of GLP-1RA liraglutide on important markers such as asprosin, body weight, blood glucose, and lipids in normal weight or overweight/obese T2DM patients to identify new targets for T2DM treatment.

## 2. Methods

### 2.1. Study design

Subjects: This study is a non-randomized, prospective observational study. From February 2021 to January 2022, 90 newly diagnosed T2DM patients according to the 2021 ADA guidelines^[10]^ were recruited from the Guangzhou Development District Hospital and the Affiliated TCM Hospital of Guangzhou University of Chinese Medicine (T2DM group). The T2DM group had a mean age of 54.91 ± 8.24 years, with 48 men and 42 women and were divided into two groups according to their BMI, normal weight (T2DM-N) and overweight or obese (T2DM-O). Overweight and obesity were defined using criteria of the Working Group on Obesity in China, overweight: BMI ≥ 24 kg/m^2^ and < 28 kg/m^2^, obesity: BMI ≥ 28 kg/m^2^.^[11]^ The control group consisted of 66 age-matched individuals with normal glucose tolerance (NGT group), with a mean age of 52.15 ± 7.97 years, 35 males and 31 females.

Exclusion criteria: age < 18 or > 70 years old; type 1 diabetes; various acute diabetic complications; acute and chronic infections, tumors, haematological disorders, rheumatic disorders, liver, kidney, heart, or brain disease; glomerular filtration rate < 45 mL/min; patients unable to tolerate adverse drug reactions and discontinue the drug; history of alcohol abuse, pregnancy, and lactation; psychiatric disorders and previous and family history; those who had used drugs for the treatment of diabetes during the past 3 months; and Refusal to cooperate.

The T2DM group was treated with 0.6 mg/d of liraglutide for the first 2 weeks, 1.2 mg/d for the subsequent 4 weeks, and 1.8 mg/d for the following 16 weeks. Various indicators of the two T2DM groups (T2DM-N and T2DM-O) were measured at baseline and after treatment (22 weeks). The NGT group was not treated with placebo or liraglutide, and various indicators were only measured at baseline. The parameters included weight, height, waist circumference, and blood pressure. Weight was measured in “kg”, height, and waist circumference were measured in “cm” to the nearest 0.1 cm, and BMI was calculated by weight (kg) ÷ height (m)^2^. Blood pressure was measured twice and then averaged to determine the final blood pressure value in mm Hg.

### 2.2. Biochemical analysis

The biomarkers included fasting glucose, postprandial glucose, glycated hemoglobin (HbA1c), blood urea nitrogen (BUN), creatinine (Cr), estimated glomerular filtration rate (eGFR), alanine aminotransferase (ALT), aspartate aminotransferase (AST), total cholesterol (TC), triglycerides (TG), LDL cholesterol, and HDL cholesterol were analyzed in the Department of Laboratory Medicine of the Guangzhou Development District Hospital and the Affiliated TCM Hospital of Guangzhou University of Chinese Medicine.

### 2.3. Asprosin assay

Blood samples (5 mL) were collected from the participants through a median cubital vein at baseline and after treatment (22 weeks) while they were fasting and two hours after breakfast into sterile vacuum tubes. The plasma and serum were separated by centrifugation for 15 minutes (3000 rpm) and 1.5 mL serum aliquots were stored at −80°C. The asprosin levels were quantified using a commercially available human asprosin ELISA kit (E15190h, EIAAB Science Inc., Wuhan, China) with an assay range of 1.56–100 ng/mL and analytical sensitivity of 0.938 ng/mL. The intra-assay and inter-assay coefficient of variation (CV) values were < 8% and < 10%.

### 2.4. Pancreatic islet function

Fasting insulin was assayed by chemiluminescence and measured in μU/ml. The Homeostatic Model Assessment of Insulin Resistance (HOMA-IR) was calculated as [fasting glucose (FPG, mmol/L) × fasting insulin (FINS, μU/ml)]/22.5.

### 2.5. Statistical analysis

The statistical evaluations were conducted utilizing SPSS 23.0 (IBM). The Kolmogorov–Smirnov test was used to determine whether the distribution was normally distributed. The data was displayed as mean standard deviation (SD). The NGT and T2DM groups were compared by the student’s *t* test. Paired student *t* tests were used to compare the statistical variances between the pre-and post-treatment periods. To analyze proportional differences, the chi-square test was used. The correlation between baseline metabolic parameters and serum asprosin levels was evaluated using the Pearson correlation coefficient. *P* < .05 were regarded as statistically significant in all two-tailed statistical tests.

### 2.6. Ethics statement

The study was approved by the Ethics Committee of the Guangzhou Development District Hospital and the Affiliated TCM Hospital of Guangzhou University of Chinese Medicine (Ethics No. KY2021-002) and conducted according to the principles embodied in the “Declaration of Helsinki” (2013). All participants clearly understood the process and purpose of this study. Consent forms were signed before the experiment.

## 3. Results

**Figure 1.**
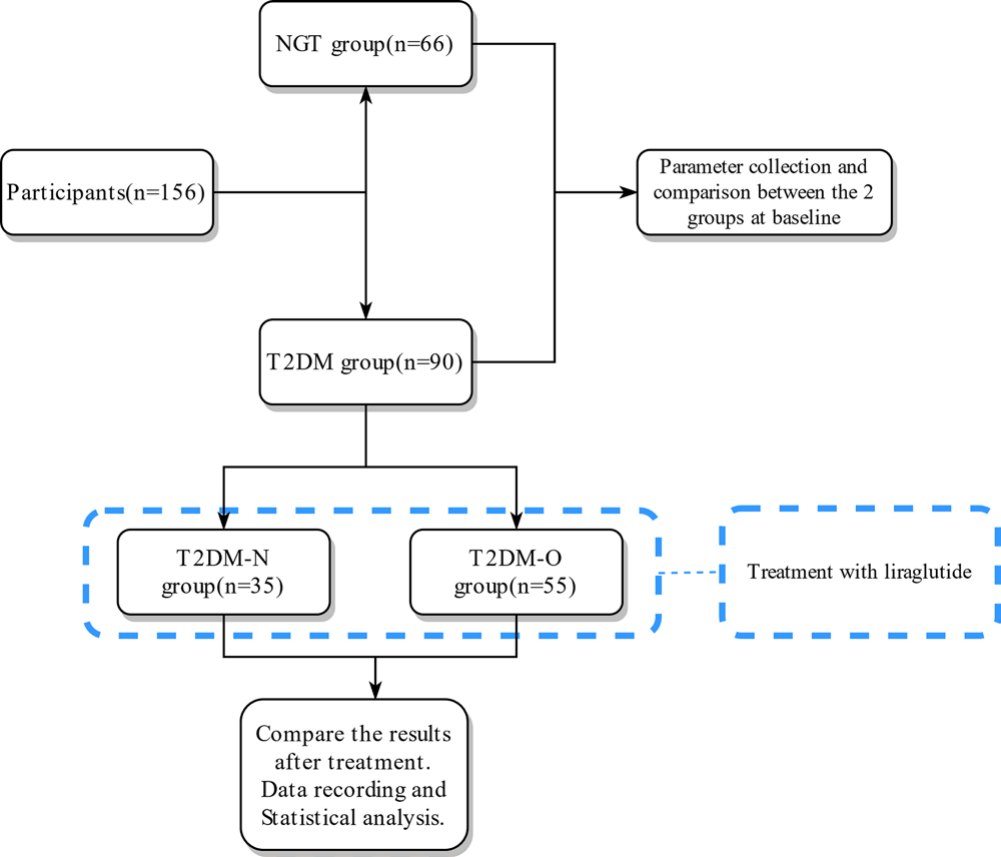
The flow diagram of the study. NGT = normalglucose tolerance, T2DM = type 2 diabetes mellitus, T2DM-N = normal weight T2DM, T2DM-O = overweight or obese T2DM.

No patients stopped treatment or withdrew from the study. Short-term nausea and diarrhea occurred in two normal weight T2DM patients and three overweight/obese T2DM patients treated with liraglutide. The flow diagram of the study was shown in Figure 1.

### 3.1. Baseline clinical characteristics

**Figure 2.**
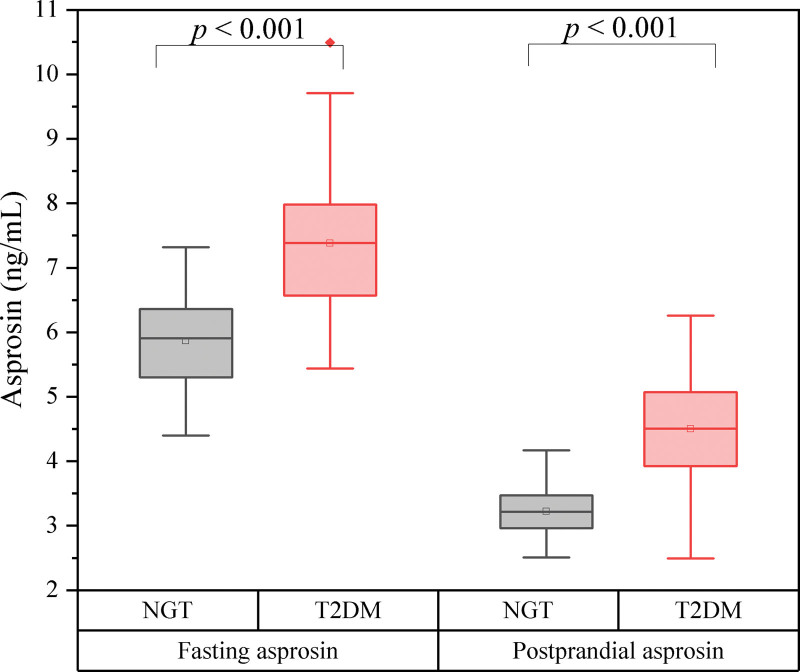
The serum fasting asprosin and postprandial asprosin levels between the T2DM group and the NGT group in baseline. NGT = normalglucose tolerance, T2DM = type 2 diabetes mellitus.

Table 1 presents the baseline clinical characteristics of the study participants showing that the T2DM and NGT groups were similar in age, sex, BMI, SBP, DBP, BUN, Cr, eGFR, AST, ALT, TC, TG, HDL-C, and LDL-C (all *P* > .05). However, the T2DM patients had significantly higher FBG, 2h postprandial glucose (2hPG), HbA1c, HOMA-IR levels than the NGT subjects (all *P* < .05). The serum fasting asprosin and postprandial asprosin levels were significantly higher in the T2DM group than in the NGT group (*P* < .001) (Fig. 2).

**Table 1:** Baseline clinical characteristics of the study participants.

Characteristics	NGT (n = 66)	T2DM (n = 90)	*t*/χ^2^	*P*
Age (yr)	52.15 ± 7.97	54.91 ± 8.24	−2.160	.032
Gender (men/women)	35/31	48/42	0.001	.970
overweight/obesity (yes/no)	36/30	55/35	0.675	.411
Body weight (kg)	64.19 ± 10.54	70.46 ± 10.34	−3.712	<.001
BMI (kg/m^2^)	24.04 ± 2.11	25.56 ± 3.44	−3.163	.002
WC (cm)	87.64 ± 6.81	95.22 ± 11.22	−4.864	<.001
SBP (mm Hg)	128.14 ± 10.16	130.88 ± 12.13	−1.490	.138
DBP (mm Hg)	76.97 ± 5.05	75.53 ± 5.55	1.658	.099
BUN (mmol/L)	4.45 ± 1.05	4.78 ± 1.22	−1.811	.072
Cr (μmol/L)	77.79 ± 17.24	74.44 ± 21.85	1.032	.304
eGFR (mL/min/1.73 m^2^)	118.21 ± 14.92	114.38 ± 13.24	1.691	.093
AST (U/L)	30.52 ± 6.64	32.39 ± 6.85	−1.711	.089
ALT (U/L)	29.92 ± 5.76	31.66 ± 8.08	−1.486	.139
FBG (mmol/L)	5.25 ± 0.55	10.16 ± 3.26	−12.118	<.001
HbA1c (%)	5.36 ± 0.43	7.61 ± 1.24	−14.207	<.001
TC (mmol/L)	4.14 ± 0.51	4.31 ± 0.81	−1.499	.136
TG (mmol/L)	1.89 ± 0.48	1.97 ± 0.64	−0.766	.445
HDL-C (mmol/L)	1.23 ± 0.31	1.14 ± 0.29	1.918	.057
LDL-C (mmol/L)	2.36 ± 0.48	2.50 ± 0.73	−1.382	0.169
2hPG (mmol/L)	6.91 ± 1.35	16.05 ± 4.53	−15.870	<.001
HOMA-IR	1.59 ± 0.41	3.40 ± 0.65	−19.758	<.001
Fasting asprosin (ng/mL)	5.87 ± 0.71	7.38 ± 1.08	−9.882	<.001
Postprandial asprosin (ng/mL)	3.22 ± 0.34	4.50 ± 0.83	−11.758	<.001

Data are presented as mean ± standard deviation or as otherwise.

2hPG = 2h postprandial glucose, ALT = alanine aminotransferase, AST = aspartate aminotransferase, BMI = body mass index, BUN = blood urea nitrogen, Cr = creatinine, DBP = diastolic blood pressure, eGFR = estimated glomerular filtration rate, FBG = fasting glucose, HbA1c = glycated hemoglobin, HDL-C = HDL cholesterol, HOMA-IR = Homeostatic Model Assessment of Insulin Resistance, LDL-C = LDL cholesterol, NGT = normal glucose tolerance, SBP = systolic blood pressure, T2DM = type 2 diabetes mellitus, TC = total cholesterol, TG = triglycerides, WC = waist circumference.

### 3.2. Correlation of asprosin with parameters at the baseline (Table 2 ) (Fig. 3 )

Correlation analysis of the T2DM group at baseline revealed that fasting asprosin levels positively correlated with BMI (*r* = 0.247, *P* = .019), FBG (*r* = 0.433, *P* < .001), 2hPG (*r* = 0.290, *P* = .006), HbA1c (*r* = 0.268, *P* = .011), TG (*r* = 0.282, *P* = .007), HOMA-IR (*r* = 0.267, *P* = .011) and was negatively correlated with HDL-C (*r* = −0.295, *P* = .005). Postprandial asprosin concentration was positively correlated with BMI (*r* = 0.269, *P* = .010), FBG (*r* = 0.429, *P* < .001), 2hPG (*r* = 0.271, *P* = .010), HbA1c (*r* = 0.299, *P* = .004), TG (*r* = 0.256, *P* = .015), HOMA-IR (*r* = 0.250, *P* = .017) and was negatively correlated with HDL-C (*r* = −0.292, *P* = .005).

**Table 2:** Correlations between serum asprosin levels and the baseline parameters.

Characteristics	NGT fasting asprosin		NGT postprandial asprosin		T2DM fasting asprosin		T2DM postprandial asprosin	
	*r*	*P*	*r*	*P*	*r*	*P*	*r*	*P*
Age	0.160	.198	0.183	.141	0.136	.203	0.125	.241
Body weight	0.134	.282	0.145	.244	0.127	.232	0.196	.065
BMI	0.252	.041	0.304	.013	0.247	.019	0.269	.010
WC	0.162	.194	0.105	.400	0.181	.088	0.174	.102
SBP	0.114	.361	0.176	.156	0.105	.327	0.066	.539
DBP	0.211	.090	0.225	.069	0.098	.360	0.084	.431
BUN	0.206	.096	0.135	.280	0.118	.268	0.066	.537
Cr	0.131	.296	0.100	.423	0.104	.329	0.116	.277
eGFR	−0.146	.242	−0.018	.888	−0.065	.544	−0.015	.887
AST	0.186	.134	0.224	.071	0.167	.116	0.207	.051
ALT	0.130	.297	0.153	.221	0.161	.129	0.150	.158
FBG	0.394	.001	0.441	<.001	0.433	<.001	0.429	<.001
2hPG	0.299	.015	0.354	.004	0.290	.006	0.271	.010
HbA1c	0.331	.007	0.346	.004	0.268	.011	0.299	.004
TC	0.237	.055	0.154	.218	0.117	.273	0.101	.344
TG	0.385	.001	0.442	<.001	0.282	.007	0.256	.015
HDL-C	−0.279	.023	−0.337	.006	−0.295	.005	−0.292	.005
LDL-C	0.168	.176	0.152	.223	0.148	.163	0.132	.213
HOMA-IR	0.303	.014	0.292	.017	0.267	.011	0.250	.017

2hPG = 2h postprandial glucose, ALT = alanine aminotransferase, AST = aspartate aminotransferase, BMI = body mass index, BUN = blood urea nitrogen, Cr = creatinine, DBP = diastolic blood pressure, eGFR = estimated glomerular filtration rate, FBG = fasting glucose, HbA1c = glycated hemoglobin, HDL-C = HDL cholesterol, HOMA-IR = Homeostatic Model Assessment of Insulin Resistance, LDL-C = LDL cholesterol, NGT = normal glucose tolerance, SBP = systolic blood pressure, T2DM = type 2 diabetes mellitus, TC = total cholesterol, TG = triglycerides, WC = waist circumference.

**Figure 3.**
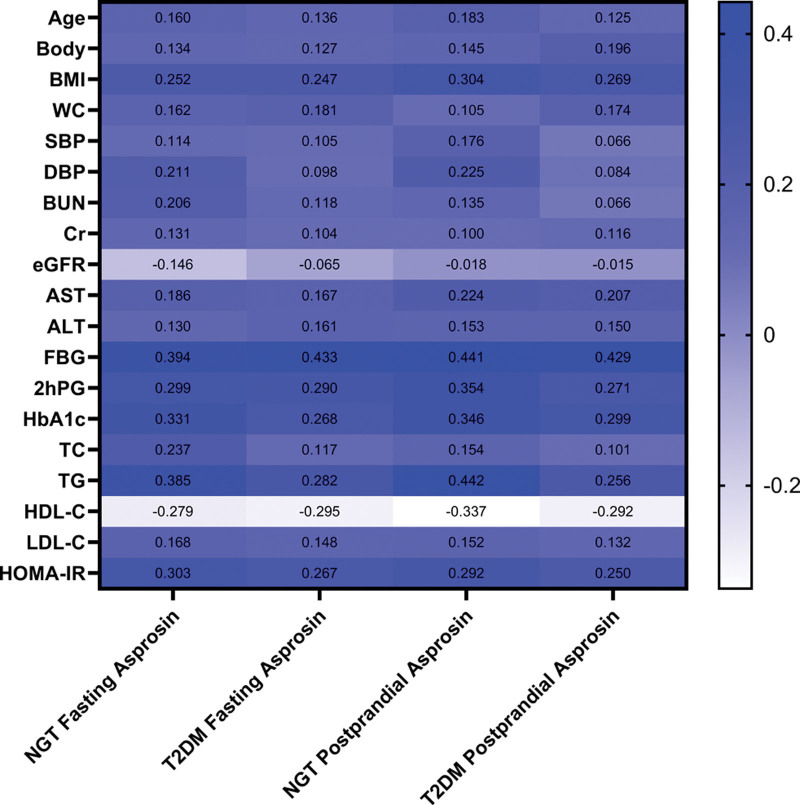
Correlations between serum asprosin levels and the baseline parameters. 2hPG = 2h postprandial glucose, ALT = alanine aminotransferase, AST = aspartate aminotransferase, BMI = body mass index, BUN = blood urea nitrogen, Cr = creatinine, DBP = diastolic blood pressure, eGFR = estimated glomerular filtration rate, FBG = fasting glucose, HbA1c = glycated hemoglobin, HDL-C = HDL cholesterol, HOMA-IR = Homeostatic Model Assessment of Insulin Resistance, LDL-C = LDL cholesterol, NGT = normal glucose tolerance, SBP = systolic blood pressure, T2DM = type 2 diabetes mellitus, TC = total cholesterol, TG = triglycerides, WC = waist circumference.

Fasting asprosin in the NGT group at baseline positively correlated with BMI (*r* = 0.252, *P* = .041), FBG (*r* = 0.394, *P* = .001), 2hPG (*r* = 0.299, *P* = .015), HbA1c (*r* = 0.331, *P* = .007), TG (*r* = 0.385, *P* = .001), HOMA-IR (*r* = 0.303, *P* = .014) and negatively correlated with HDL-C (*r* = −0.279, *P* = .023). Postprandial asprosin positively correlated with BMI (*r* = 0.304, *P* = .013), FBG (*r* = 0.441, *P* < .001), 2hPG (*r* = 0.354, *P* = .004), HbA1c (*r* = 0.346, *P* = .004), TG (*r* = 0.442, *P* < .001), HOMA-IR (*r* = 0.292, *P* = .017) and negatively correlated with HDL-C (*r* = −0.337, *P* = .006)

### 3.3. Comparison of biochemical parameters before and after treatment with liraglutide (Table 3 )

**Figure 4.**
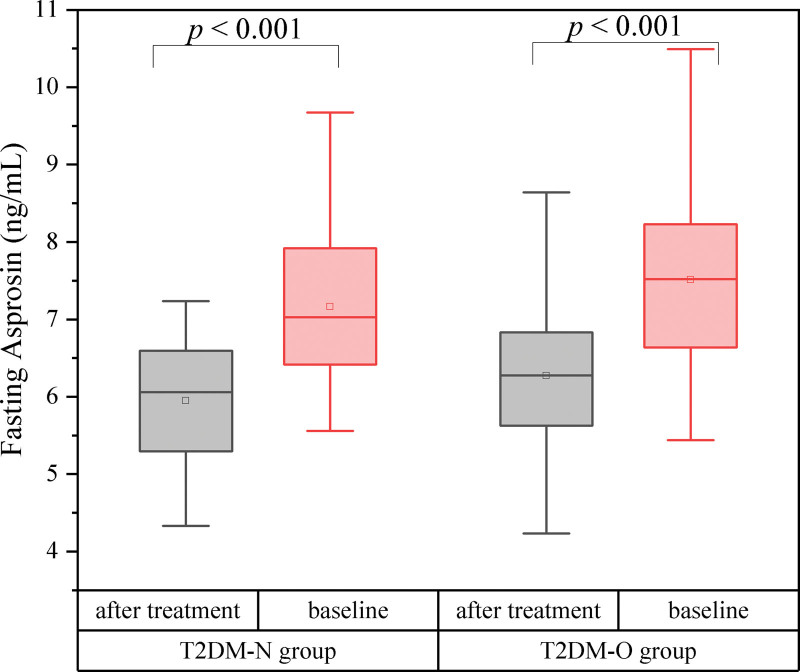
Fasting asprosin levels between after treatment and before treatment. T2DM-N = normal weight T2DM, T2DM-O = overweight or obese T2DM.

**Figure 5.**
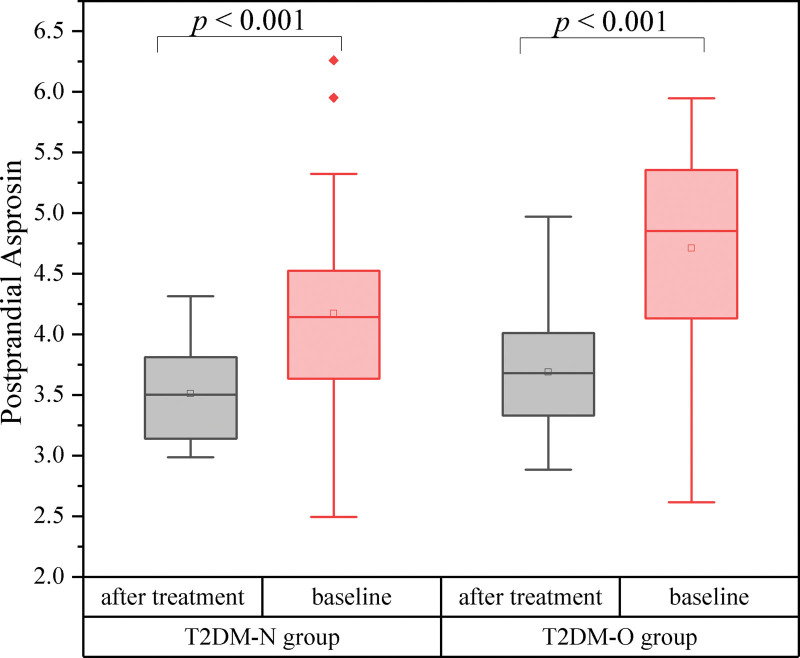
Postprandial asprosin levels between after treatment and before treatment. T2DM-N = normal weight T2DM, T2DM-O = overweight or obese T2DM.

WC, fasting glucose, postprandial glucose, HbA1c, TC, TG, LDL-C, HOMA-IR, and asprosin levels were reduced, while HDL cholesterol was elevated after liraglutide treatment compared to pretreatment in the normal weight group with T2DM (T2DM-N group) (*P* < .05) (Fig. 4). Likewise, weight, BMI, WC, fasting glucose, postprandial glucose, HbA1c, TC, TG, LDL-C, HOMA-IR, and asprosin levels decreased, while HDL cholesterol increased after liraglutide treatment compared to baseline in the overweight/obesity group with T2DM (T2DM-O group) (*P* < .05) (Fig. 5).

**Table 3:** Comparison of biochemical parameters before and after treatment with liraglutide in the type 2 diabetes group.

Characteristics	T2DM-N (n = 35)				T2DM-O (n = 55)			
	Before treatment	After treatment	*t*	*P*	before treatment	after treatment	t	*P*
Body weight (kg)	62.08 ± 7.43	59.84 ± 5.89	1.374	.179	75.79 ± 8.18	70.03 ± 7.02	3.968	<.001
BMI (kg/m^2^)	21.95 ± 1.56	21.39 ± 1.67	1.415	.166	27.85 ± 2.03	26.11 ± 2.11	4.440	<.001
WC (cm)	84.04 ± 4.13	81.95 ± 4.55	2.194	.035	102.33 ± 8.02	96.36 ± 7.52	3.746	<.001
SBP (mm Hg)	130.34 ± 15.57	129.77 ± 11.47	0.164	.871	130.67 ± 8.74	129.82 ± 10.30	0.445	.658
DBP (mm Hg)	78.69 ± 5.02	77.43 ± 5.83	0.840	.407	73.53 ± 4.94	74.60 ± 4.28	−1.176	.245
BUN (mmol/L)	4.49 ± 1.35	4.34 ± 1.45	0.499	.621	4.97 ± 1.11	4.75 ± 0.98	1.028	.308
Cr (μmol/L)	75.57 ± 21.11	67.54 ± 15.90	1.775	.085	73.72 ± 22.47	71.23 ± 18.98	0.594	.555
eGFR (mL/min/1.73 m^2^)	114.58 ± 10.32	117.67 ± 11.79	−1.246	.221	114.25 ± 14.89	114.73 ± 12.62	−0.180	.858
AST (U/L)	32..01 ± 4.76	29.78 ± 6.48	1.849	.073	32.63 ± 7.94	29.98 ± 8.23	1.676	.100
ALT (U/L)	29.24 ± 7.15	28.17 ± 6.75	0.580	.566	33.20 ± 8.32	32.94 ± 9.28	0.143	.887
FBG (mmol/L)	9.33 ± 2.73	6.85 ± 0.83	5.066	<.001	10.69 ± 3.48	7.14 ± 0.99	7.227	<.001
2hPG (mmol/L)	15.48 ± 3.84	10.00 ± 2.26	7.886	<.001	16.42 ± 4.92	10.33 ± 2.38	9.399	<.001
HbA1c (%)	7.72 ± 1.54	5.90 ± 1.05	5.117	<.001	7.54 ± 1.00	6.07 ± 1.10	7.141	<.001
TC (mmol/L)	4.07 ± 0.78	3.53 ± 0.60	3.52	.001	4.46 ± 0.80	3.61 ± 0.64	5.647	<.001
TG (mmol/L)	1.81 ± 0.38	1.37 ± 0.37	4.086	<.001	2.07 ± 0.74	1.82 ± 0.41	2.174	.034
HDL-C (mmol/L)	1.10 ± 0.31	1.87 ± 0.39	−8.457	<.001	1.16 ± 0.28	1.59 ± 0.29	−8.295	<.001
LDL-C (mmol/L)	2.16 ± 0.62	1.82 ± 0.63	2.129	.041	2.72 ± 0.72	2.21 ± 0.49	4.622	<.001
HOMA-IR	3.21 ± 0.59	2.25 ± 0.41	7.520	<.001	3.51 ± 0.67	2.45 ± 0.57	10.933	<.001
Fasting asprosin (ng/mL)	7.17 ± 0.97	5.95 ± 0.80	5.144	<.001	7.51 ± 1.13	6.27 ± 0.89	6.600	<.001
Postprandial asprosin (ng/mL)	4.17 ± 0.78	3.51 ± 0.36	4.373	<.001	4.71 ± 0.81	3.69 ± 0.46	8.953	<.001

Data are presented as mean ± standard deviation.

2hPG = 2h postprandial glucose, ALT = alanine aminotransferase, AST = aspartate aminotransferase, BMI = body mass index, BUN = blood urea nitrogen, Cr = creatinine, DBP = diastolic blood pressure, eGFR = estimated glomerular filtration rate, FBG = fasting glucose, HbA1c = glycated hemoglobin, HDL-C = HDL cholesterol, HOMA-IR = Homeostatic Model Assessment of Insulin Resistance, LDL-C = LDL cholesterol, SBP = systolic blood pressure, T2DM-N = type 2 diabetes mellitus with normal weight, T2DM-O = type 2 diabetes mellitus with overweight/obese, TC = total cholesterol, TG = triglycerides, WC = waist circumference.

## 4. Discussion

This study found that the T2DM group had more abnormal metabolic markers than NGT controls. Also, serum asprosin concentrations were higher in T2DM than in those with NGT, both in the fasting and postprandial states, in line with previous research. Some studies^[12]^ have shown that fasting and postprandial asprosin levels are higher in T2DM individuals than in those with NGT and that fasting asprosin levels are strongly associated with T2DM. Another study compared 84 adults with T2DM to 86 people with NGT showing that the adults with T2DM had considerably higher serum asprosin concentrations than controls. Therefore, asprosin may be a risk factor for T2DM pathogenesis.^[13]^

The present study also revealed a positive correlation between fasting asprosin or postprandial asprosin and BMI, 2hPG, HbA1c, TG, HOMA-IR, and a negative correlation with HDL-C, broadly in line with previous studies.^[14,15]^ However, unlike previous reports, no correlation between asprosin and waist circumference was found in this study. Considering that height and weight vary among individuals, waist circumference values alone may not be a true reflection of the degree of obesity, which may account for the lack of correlation between asprosin and waist circumference. Another potential reason is ethnic variability, as Asians have a high visceral fat content but do not have a prominent waist circumference.^[16]^

WC, fasting glucose, postprandial glucose, HbA1c, TC, TG, LDL-C, HOMA-IR, and asprosin concentrations decreased and HDL-C increased after liraglutide treatment in both normal weight or overweight/obesity patients with T2DM compared to baseline. In particular, weight and BMI decreased in obese patients with T2DM. Consistent with previous studies, liraglutide reduced WC, glucose, lipid, and asprosin levels in patients with T2DM.

GLP-1 is influenced by feeding activity. It is released from the intestinal tissues into the blood immediately after a meal and promotes insulin secretion, increasing glucose uptake and glycogen synthesis and reducing glucagon production. It delays gastric emptying, making people feel fuller and less hungry, so effectively reduces body mass in obese diabetics as well as improves blood glucose levels in diabetics.^[17]^

GLP-1RA, particularly liraglutide, facilitates weight loss by suppressing appetite, reducing food intake, significantly increasing the level of satiety signals in the hypothalamic arcuate nucleus, and inhibiting the increase in hunger signals in the arcuate nucleus, thereby increasing satiety and reducing caloric intake; increasing energy expenditure, promoting the conversion of visceral white fat to brown fat, and promoting brown fat thermogenesis; and affecting gastrointestinal motility, prolonging gastric emptying time, and reducing gastric acid secretion stimulated by pentagastrin. It is important to note that it has little effect on weight loss in people with low-fat content.^[18]^

In this study, glucose and HbA1c control in both T2DM groups (T2DM-N and T2DM-O) were better after treatment. Insulin resistance was also alleviated. Weight and BMI in obese type 2 diabetic patients decreased dramatically with treatment. Clinical trials have shown that liraglutide helps with weight loss. A single-center study in Belgium showed that obese patients treated with liraglutide for 4 months had considerable weight loss regardless of the maximum tolerated maintenance dose. Weight loss was significant even when the maximum daily dose was not reached.^[19]^

However, despite a decrease in body weight and BMI in the normal weight T2DM group after treatment, the difference was not statistically significant. Liraglutide may have a limited effect on weight loss in normal weight individuals. More trials are needed to confirm this.

Asprosin was decreased in T2DM patients after liraglutide treatment, which is consistent with previous studies. The reason may be the hypolipidemic effect of liraglutide. Because asprosin is synthesized from adipose tissue, asprosin may decrease with the reduction of adipose tissue. This needs to be further verified. Liraglutide has a direct stimulatory effect on Pro-opiomelanocortin (POMC) neurons and inhibits Agouti-related peptide (AgRP) neurons, lowering appetite and increasing satiety.^[20]^ In contrast, asprosin crosses the blood-brain barrier and activates pro-appetitive AgRP neurons, and inhibits anorexic POMC neurons via the G protein-cAMP-PKA pathway.^[21]^ Liraglutide may antagonize the neuronal activity of asprosin, thereby suppressing appetite and reducing body weight.

Some studies have demonstrated higher asprosin concentrations in people with abnormal blood glucose. Asprosin deficiency may lead to loss of appetite and extreme leanness.^[22]^ Diminished asprosin activity or asprosin depletion could be an innovative therapeutic modality for the treatment of T2DM and obesity, thus a potential target for diabetes treatment.^[23]^

Liraglutide can reduce SBP compared to placebo, however, when the intervention lasted more than 1 year, the difference was no longer significant.^[24]^ Furthermore, the effect of liraglutide on blood pressure was dose-dependent. A study in patients with coronary artery disease with abnormal blood glucose showed that liraglutide had no significant effect on blood pressure.^[25]^ Another study evaluated the effectiveness of dulaglutide versus liraglutide, with only dulaglutide significantly lowering systolic blood pressure after 12 months.^[26]^ In our study, liraglutide did not affect SBP and the reason for this discrepancy in studies needs to be confirmed.

Few studies have investigated the effects of liraglutide on asprosin. In this study, T2DM patients were separated into two groups based on their weight, normal weight or overweight/obese to evaluate the effects of liraglutide on waist circumference, body mass, BMI, and asprosin. Additionally, only a few studies have observed postprandial asprosin before and after drug treatment. The present study specifically examined the changes in postprandial asprosin before and after treatment, as well as exploring the correlation between postprandial asprosin at baseline and indicators such as body weight, blood glucose, and blood lipids.

### 4.1. Limitations

This study has several limitations. The sample size was relatively small and further larger studies should be performed. In addition, lifestyle interventions are a cornerstone of first-line T2DM treatment, so the impact of lifestyle changes on the effectiveness of liraglutide treatment cannot be excluded. Studies have shown that exercise can reduce asprosin levels^.[27,28]^ Factors such as exercise and dietary patterns were not included in our study, so we cannot exclude the effect of lifestyle changes on asprosin. We did not systematically evaluate lifestyle changes, so we cannot rule out the impact of lifestyle changes on asprosin. Physical activities and dietary modifications could be included in the follow-up studies. Nevertheless, as a preliminary study, the findings are relevant to clinical practice.

## 5. Conclusion

In conclusion, fasting and postprandial asprosin concentrations in T2DM are higher than in those with NGT and fasting asprosin and postprandial asprosin positively correlated with BMI, 2hPG, HbA1c, TG, HOMA-IR, and negatively correlated with HDL-C. WC, FBG, postprandial glucose, HbA1c, TC, TG, LDL-C, HOMA-IR, and asprosin decreased and HDL increased in T2DM patients after liraglutide treatment, in particular, liraglutide caused a considerable decrease in body weight and BMI in overweight/obese T2DM patients. The precise mechanisms by which the GLP1-RA decreases the level of asprosin and how liraglutide may antagonize these mechanisms remain to be clarified. It can be assumed that liraglutide may antagonize asprosin activity thus inhibiting appetite and causing weight loss but further studies are needed to elucidate these complex signaling mechanisms.

## Author contributions

**Conceptualization:** Chenggang Dai, Weifeng Zhu.

**Data curation:** Chenggang Dai.

**Formal analysis:** Chenggang Dai.

**Investigation:** Chenggang Dai.

**Methodology:** Chenggang Dai, Weifeng Zhu.

**Resources:** Chenggang Dai, Weifeng Zhu.

**Software:** Chenggang Dai.

**Supervision:** Weifeng Zhu.

**Writing – original draft:** Chenggang Dai.

**Writing – review & editing:** Weifeng Zhu.
